# Implantable device measured objective daily physical activity as a predictor of long-term all-cause mortality and cardiac death in patients with age > 75 years and high risk of sudden cardiac death: a cohort study: Physical activity and patients over 75 years old

**DOI:** 10.1186/s12877-022-02813-1

**Published:** 2022-02-16

**Authors:** Xiaoyao Li, Keping Chen, Wei Hua, Yangang Su, Jiefu Yang, Zhaoguang Liang, Wei Xu, Shuang Zhao, Zeyi Li, Shu Zhang

**Affiliations:** 1 Arrhythmia Center, State Key Laboratory of Cardiovascular Disease, Fuwai Hospital, National Center for Cardiovascular Diseases, Chinese Academy of Medical Sciences and Peking Union Medical College, 167 Bei Li Shi Road, Xicheng District, Beijing, 100037 China; 2 Department of Cardiology, Shanghai Institute of Cardiovascular Diseases, Zhongshan Hospital, Fudan University, Shanghai, China; 3 Department of Cardiology, Beijing Hospital, Beijing, China; 4 Department of Cardiology, First Affiliated Hospital of Harbin Medical University, Harbin, China; 5 Department of Cardiology, Nanjing Drum Tower Hospital, Nanjing, China

**Keywords:** Patients over 75 years old, Sudden cardiac death, Implantable device measured objective physical activity, All-cause mortality, Cardiac death

## Abstract

**Background:**

To study the relationship between objective daily physical activity (PA), as measured by implantable cardioverter defibrillators (ICDs)/cardiac resynchronization therapy defibrillators (CRTDs), and long-term prognoses in patients with age > 75 years at high risk of sudden cardiac death (SCD).

**Methods:**

In total, 133 patients with age > 75 years old (age 79.52 ± 3.68 years) in the SUMMIT study were retrospectively analysed. The major endpoint was all-cause mortality, and the minor endpoint was cardiac death.

**Results:**

The mean follow-up time was 57.1 ± 24.2 months (range: from 4 to 96 months). In total, 46 all-cause mortality and 23 cardiac death events occurred. The receiver operating characteristic curve indicated a baseline PA cut-off value of 6.47% (93 min/day) can predict all-cause mortality in patients with age > 75 years, with an area under the curve of 0.670 (95% confidence interval (CI): 0.573–0.767, *P* = 0.001). The sensitivity was 67.4%, and the specificity was 66.7%. Patients with baseline PA ≤ 6.47% had higher rates of all-cause mortality (51.7% vs 20.5%, *P* < 0.001) and cardiac death (25.0% vs 11.0%, *P* = 0.040). The estimated Kaplan-Meier survival curves showed that patients with PA ≤ 6.47% had an increased cumulative incidence of all-cause mortality (Log-rank *P* < 0.0001) and cardiac death (Log-rank *P* = 0.0067). Multivariate Cox regression analysis showed that PA ≤ 6.47% was an independent predictor of all-cause mortality (hazard ratio (HR) 3.137, 95% CI: 1.667–5.904, *P* < 0.001) and cardiac death (HR value 3.345, 95% CI: 1.394–8.028, *P* = 0.007).

**Conclusions:**

Daily PA of about 1.5 h was associated with lower all-cause mortality and cardiac death risk in patients with age > 75 years and high risk of SCD with ICDs/CRTDs. PA monitoring may aid in long-term management of older patients at high risk of SCD.

## Background

The global population is ageing rapidly, leading to an increased burden on the health and economic systems [1]. Cardiovascular disease has been the major cause of mortality in older people [2]. Sudden cardiac death (SCD), as a serious public health problem worldwide, accounts for virtually half of all cardiovascular deaths [3]. An implantable cardioverter defibrillator (ICD) can effectively terminate malignant tachyarrhythmia, prevent SCD and improve survival rate [4]. A number of studies indicate physical activity (PA) is related to many chronic diseases, premature mortality, and poor cardiovascular prognoses [5, 6]. However, PA is a safe, low-cost, environmentally friendly, easily accessible treatment strategy that is often not implemented in clinical practice. For older people in particular, physical activities are often limited. Survey results have shown that a low proportion of patients, especially older patients, perform the recommended amount of PA [7, 8].

Most of the previous studies on PA and cardiovascular diseases used self-assessment questionnaires, which have a certain level of bias and error, such as recall biases, especially for older people due to their levels of education and cognitive function [9, 10]. In addition, most of the populations in previous studies generally comprised middle-aged adults. Studies have shown that PA recorded by remote cardiovascular implantable electronic devices (CIEDs) is related to cardiovascular prognoses [11]. With the extension of life expectancy and continuous advancements in CIED implantation technology, an increasing number of old people undergo CIED implantation, and the proportion of old people implanted with CIEDs is also increasing. The relationship between PA and cardiovascular prognoses patients with age > 75 years and implantable cardioverter defibrillators (ICDs)/cardiac resynchronization therapy defibrillators (CRTDs) is not well established.

In the present study, continuous PA was recorded by ICDs/CRTDs, and its correlation with all-cause mortality and cardiac death in old population over 75 years old was investigated.

## Methods

### Population

Home monitoring (HM) transmission data archived between June 2010 and August 2014 from the SUMMIT registry (Study of Home Monitoring System Safety and Efficacy in Cardiac Implantable Electronic Device-Implantable Patients) in China were retrospectively analysed. For continuous patient monitoring, the HM setting on all devices was turned “on”. The present study complied with the principles of the Declaration of Helsinki and was approved by ethics committee of Fuwai Hospital (the chief institute) and all other participating organizations (Zhongshan Hospital Fudan University et al.). All patients signed informed consent forms before enrollment.

Among those participants, patients with an age older than or equal to 75 years, eligibility for an ICD or a CRTD according to the recommended indications, an implanted ICD/CRTD (Biotronik, Germany) device with HM and surviving more than 3 months after the device implantation were selected. While patients who could not be followed up or had missing HM data, patients with neoplastic diseases or a life expectancy of less than 1 year or patients with disabilities and other diseases that restricted their daily activities were excluded.

### Baseline characteristics

Baseline characteristics for all admitted patients in this study were obtained from the patients’ medical records before implantation, including demographic characteristics (age, sex, body mass index (BMI), the New York Heart Association (NYHA) class, comorbidities (ischemic cardiomyopathy, hypertension, diabetes (DM), atrial fibrillation (AF), stroke, syncope), echocardiographic indices (left ventricular ejection fraction (LVEF), left ventricular end diastolic diameter (LVEDD), and medication (renin angiotensin system blocker, beta blocker, diuretic, and amiodarone). All data were obtained and assesed by two independent physicians who were blinded to the other results.

### PA definition and measurement

PA in ICD/CRT-D was measured through an integrated circuit accelerometer embedded in the pulse generator. PA was measured as the time during which the Biotronik devices’ motion sensors delivered rates higher than the devices’ basic rates. The threshold value judged as “active” was corresponding to 2 metabolic equivalents (METs) [11, 12]. The percentage of active time per day was recorded as the daily PA; for example, 10% PA indicated 2.4 h of daily PA. The Biotronik remote monitoring system automatically transmitted the data stored in the implantable devices to the Biotronik service centre every day.

As the level of PA after implantation was considered to be less than usual, the data were collected during the first 30–60 days after ICD/CRTD implantation, in accordance with the previous study, and the mean value of the 30-day PA data was used as the baseline PA for each patient.

### Follow-up and endpoints

Follow-up was conducted regularly for all patients enrolled. If the patient’s daily transmission was interrupted, the clinical research coordinator immediately contacted his or her family to confirm the patient’s condition. The primary endpoint of this study was all-cause mortality, and the secondary endpoint was cardiac death. The cause of death was obtained by contact with the family and was determined by the death certificate.

### Statistical methods

Software SPSS Statistics version 23.0 (IBM Corp., Armonk, New York) and GraphPad Prism software version 7.0 (GraphPad Software, La Jolla, California) were used to perform all statistical analysis. Continuous variables were presented as the mean ± SD and compared using Student’s t-test of variance. Categorical data were presented as numbers (percentages) and compared with Chi-square tests. Dot plot was conducted to compare PA levels between patients who died and survived. Receiver operating characteristic curves were plotted to obtain a cut-off value for quantitative variables. The categories of PA ≤ 6.47% and PA > 6.47% were used for the calculations performed. We performed Kaplan-Meier curves to compare the difference between groups regarding the endpoints and the Log-rank test was used. Then Cox proportional hazard models were used and all variables that had a statistically significant effect were introduced into a multivariate Cox proportional hazards model. Hazard ratios (HRs) and 95% confidence intervals (CIs) were calculated to determine the impact. Two-sided *P* value < 0.05 was considered significant in all tests.

## Results

### Baseline characteristics

**Fig. 1:**
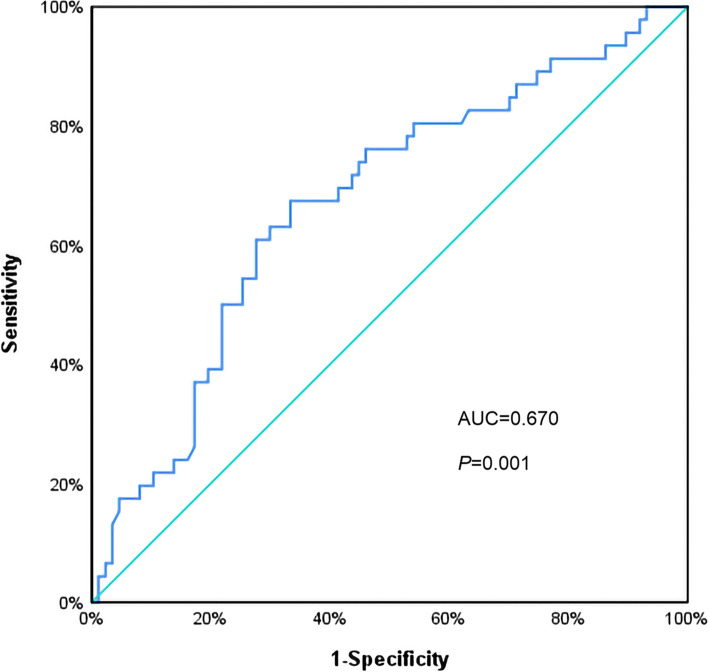
ROC curve with a cut-off value of 6.47% for PA to predict all-cause mortality, *P* = 0.001. Abbreviations: ROC curve: receiver operating characteristic curve. PA, physical activity

In total, 133 patients over 75 years old with ICDs/CRTDs were included in the present study. The average age was 79.52 ± 3.68 years old. The study cohort predominantly comprised males (74.4%). The mean baseline PA was 7.85% ± 4.64%. Receiver operating characteristic curve analysis determined that a PA cut-off value of 6.47% (93 min/day) can predict all-cause mortality. The area under the curve was 0.670 (95% CI: 0.573–0.767, *P* = 0.001), with a sensitivity of 67.4% and a specificity of 66.7% (Fig. 1).

All eligible patients were grouped by the PA cut-off value. Comparisons of patients’ baseline characteristics are presented in Table 1. Patients whose PA ≤ 6.47% had poorer NYHA classifications, had more cases of DM, and had taken more diuretics, although the difference did not reach statistical significance. There were no differences between the two groups in the other baseline characteristics.

**Table 1:** Baseline characteristics according to PA

	Total (*N* = 133)	PA ≤ 6.47% (*n* = 60)	PA > 6.47% (*n* = 73)	*P-value*
**Demographics**				
Age, years	79.52 ± 3.68	79.86 ± 3.76	79.23 ± 3.62	0.325
Male, n(%)	99 (74.4)	46 (76.7)	53 (72.6)	0.691
BMI, Kg/m2	23.17 ± 2.90	23.42 ± 2.93	23.00 ± 2.87	0.371
NYHA III/IV, n(%)	83 (62.4)	42 (50.6)	41 (49.4)	0.109
PA,%	7.85 ± 4.64	3.99 ± 1.77	11.03 ± 3.77	< 0.001
CRT-D, n(%)	34 (25.6)	17 (28.3)	17 (23.3)	0.553
Primary prevention, n(%)	66 (49.6)	29 (48.3)	37 (50.7)	0.862
**Comorbidities**				
ICM, n(%)	69 (51.9)	32 (53.3)	37 (50.7)	0.862
HTN, n(%)	62 (46.6)	24 (40.0)	38 (52.1)	0.221
DM, n(%)	21 (15.8)	14 (23.3)	7 (9.6)	0.034
Stroke, n(%)	5 (3.8)	3 (5.0)	2 (2.7)	0.657
AF, n(%)	17 (12.8)	8 (13.3)	9 (12.3)	1.000
Syncope, n(%)	27 (20.3)	14 (23.3)	13 (17.8)	0.517
**Echocardiography**				
LVEF, %	42.00 ± 13.53	41.90 ± 14.25	42.09 ± 13.00	0.936
LVEDD, mm	58.13 ± 11.62	56.47 ± 10.13	59.50 ± 12.61	0.134
**Medication**				
ACEI/ARB, n(%)	55 (41.4)	27 (45.0)	28 (38.4)	0.482
β-blocker, n(%)	70 (52.6)	29 (48.3)	41 (56.2)	0.388
Amiodarone, n(%)	39 (29.3)	18 (30.0)	21 (28.8)	1.000
Diuretic, n(%)	53 (39.8)	28 (46.7)	25 (34.2)	0.159

Values are expressed as the mean ± SD or n (%)

*Abbreviations*: *ACEI* Angiotensin-converting enzyme inhibitor, *AF* Atrial fibrillation, *ARB* Angiotensin receptor blocker, *BMI* Body Mass Index, *CRT-D* Cardiac resynchronization therapy defibrillator, *DM* Diabetes mellitus, *HTN* Hypertension, *ICD* Implantable cardioverter-defibrillator, *ICM* Ischemic cardiomyopathy, *LVEDD* Left ventricular end-diastolic dimension, *LVEF* Left ventricular ejection fraction, *NYHA class* New York Heart Association class, *PA* Physical activity

### Clinical outcomes

**Fig. 2:**
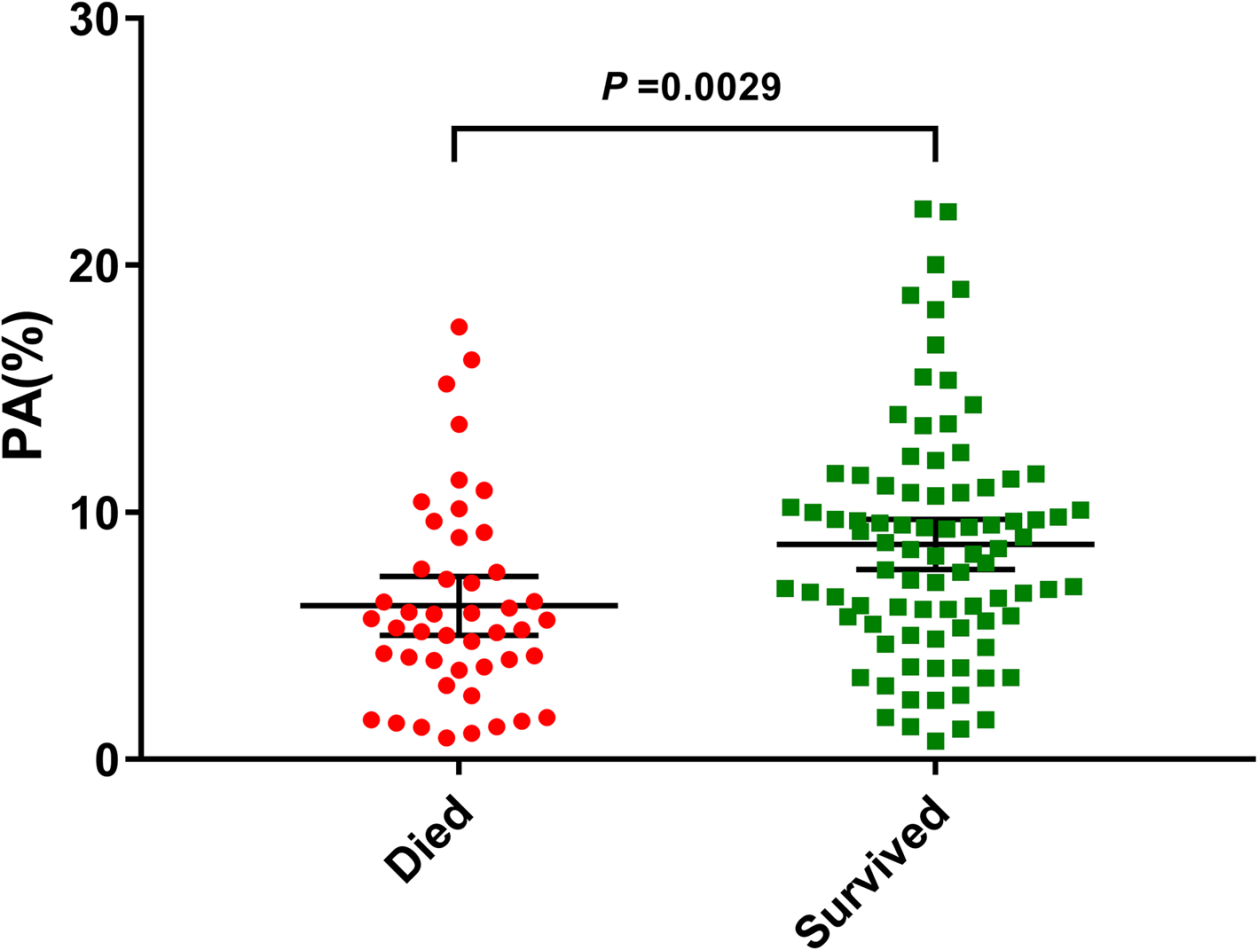
Comparison of PA levels between patients who died and survived (*P* = 0.0029). Abbreviations: PA, physical activity

The average follow-up period was 57.1 ± 24.2 months (range: from 4 to 96 months). In total, 46 all-cause deaths (34.6%) and 23 cardiac deaths (17.3%) occurred. Dot plot was conducted and the result showed that PA levels were significantly higher in patients who survived [8.51 (5.7–10.9)%] than in those died [5.48(3.82–7.68)%], *P* = 0.0029 (Fig. 2). Patients with baseline PA ≤ 6.47% had higher rates of all-cause mortality (51.7% vs 20.5%, *P* < 0.001) and cardiac death (25.0% vs 11.0%, *P* = 0.040) (Table 2). Moreover, baseline PA ≤ 6.47% was also significantly associated with non-cardiac death (26.7% vs 9.6%, *P* = 0.01).

**Table 2:** Clinical outcomes of patients depended on PA

Endpoints	Total (*N* = 133)	PA ≤ 6.47% (*n* = 60)	PA > 6.47% (*n* = 73)	*P-value*
All-cause mortality, n(%)	46 (34.6)	31 (51.7)	15 (20.5)	< 0.001
Cardiac death, n(%)	23 (17.3)	15 (25.0)	8 (11.0)	0.040

Values are expressed as n (%)

### Kaplan-Meier survival curves

**Fig. 3:**
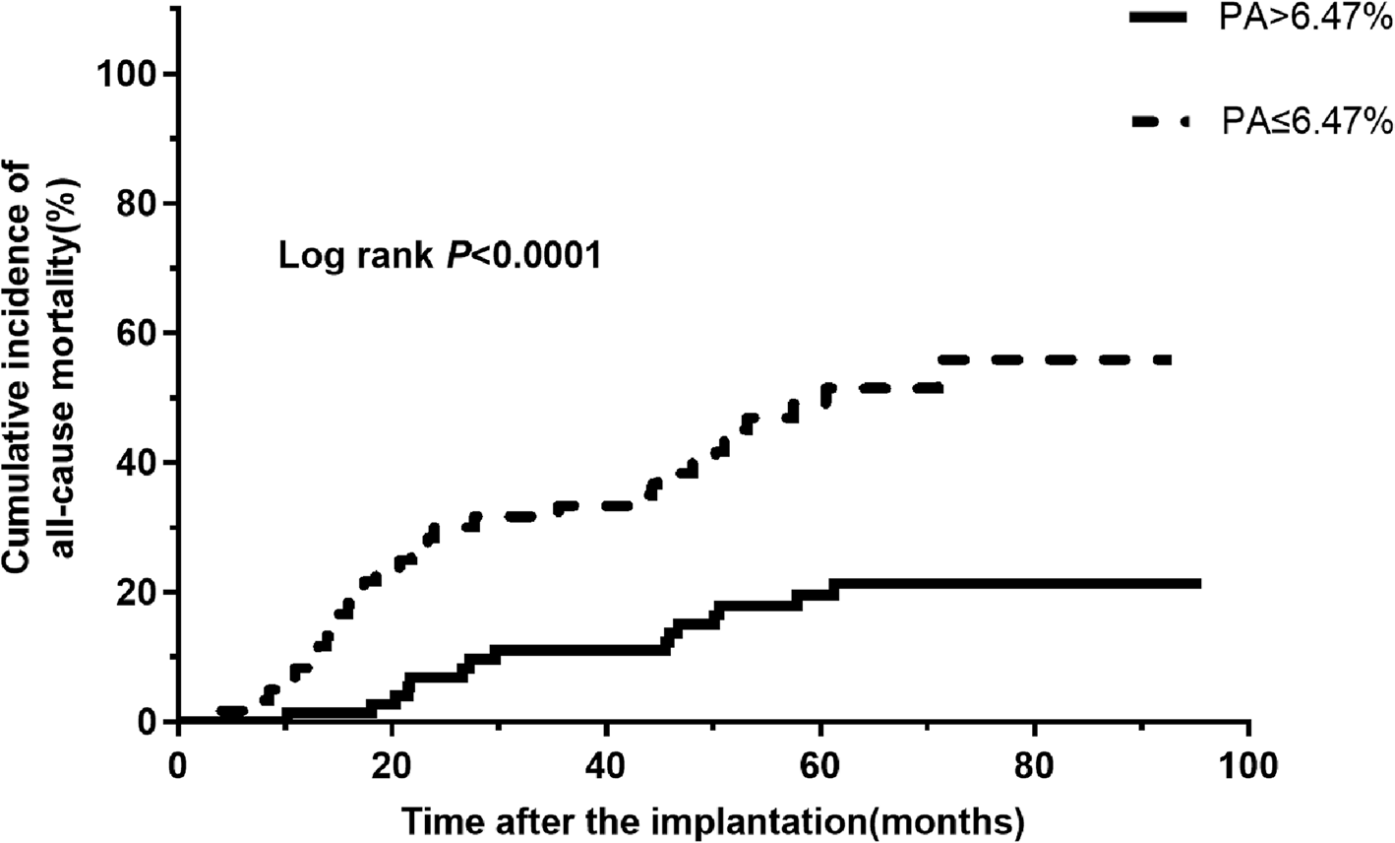
Kaplan-Meier curves for all-cause mortality in patients over 75 years with ICDs/CRTDs (Log rank *P* < 0.0001). Abbreviations: CRT-D, cardiac resynchronization therapy defibrillator; ICD, implantable cardioverter-defibrillator

**Fig. 4:**
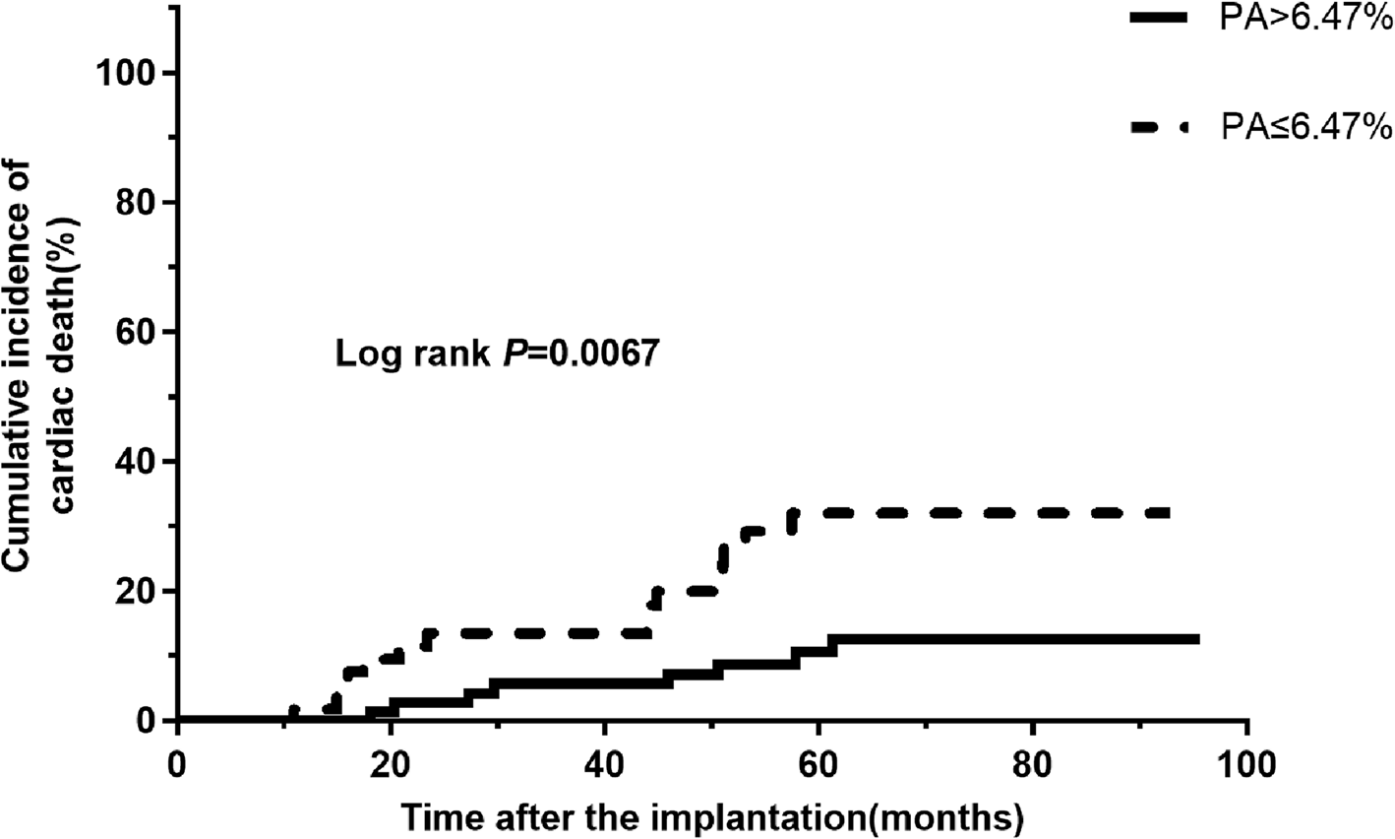
Kaplan-Meier curves for cardiac death in patients over 75 years with ICDs/CRTDs (Log rank *P* = 0.0067). Abbreviations: CRT-D, cardiac resynchronization therapy defibrillator; ICD, implantable cardioverter-defibrillator

The estimated Kaplan-Meier survival curves showed that patients with PA ≤ 6.47% had an increased cumulative incidence of all-cause mortality (Log-rank *P* < 0.0001) (Fig. 3) and cardiac death (Log-rank *P* = 0.0067) (Fig. 4).

### PA is a predictor of cardiac death and all-cause mortality

In the univariate Cox regression models, PA ≤ 6.47% was significantly related to all-cause mortality (HR 3.297, 95% CI: 1.777–6.118, *P* < 0.001). After the model was adjusted for confounders, including age, sex, LVEF, the presence of ischaemic cardiomyopathy, the presence of diabetes, the presence of AF and β-blocker consumption, the multivariate Cox regression modelling results showed that PA ≤ 6.47% was an independent risk factor for all-cause mortality (HR 3.137, 95% CI: 1.667–5.904, *P* < 0.001) (Table 3).

**Table 3:** Uni-variate and multivariate Cox analysis for endpoints

Endpoints	Uni-variate		Multivariate	
	HR(95% CI)	*P*-value	HR(95% CI)	*P*-value
All-cause mortality^a^	3.297 (1.777–6.118)	< 0.001	3.137 (1.667–5.904)	< 0.001
Cardiac death^b^	3.095 (1.310–7.310)	0.010	3.345 (1.394–8.028)	0.007

*Abbreviations*: *AF* Atrial fibrillation, *LVEF* Left ventricular ejection fraction

^a^adjusted for age, gender, LVEF, ischemic cardiomyopathy, diabetes, AF, β-blocker;

^b^adjusted for age, gender, LVEF, ischemic cardiomyopathy, AF, β-blocker

Additionally, PA ≤ 6.47% was found to be positively associated with cardiac death (HR 3.095, 95% CI: 1.310–7.310, *P* = 0.010). After the model was adjusted for confounders, including age, sex, LVEF, the presence of ischaemic cardiomyopathy, the presence of AF and β-blocker consumption, the multivariate Cox regression modelling results showed that PA ≤ 6.47% was an independent risk factor for cardiac death (HR value 3.345, 95% CI: 1.394–8.028, *P* = 0.007) (Table 3).

## Discussion

The results of the present study indicated that objective daily PA measured by the continuous remote monitoring of ICDs/CRTDs is related to the prognosis of cardiovascular diseases in patients with age > 75 years. PA ≤ 6.47% (93 min/day) was significantly associated with the risk of all-cause mortality and cardiac death. Even in patients with age > 75 years and structural heart diseases and a high risk of sudden cardiac death, daily physical activities should be performed regularly. PA monitoring may aid in long-term management of older patients at high risk of SCD.

The present study confirmed there is a correlation between PA and cardiovascular prognoses, which is consistent with the results presented in previous studies. Lear conducted a prospective cohort study in 130,000 participants from 17 countries and demonstrated that higher levels of recreational and non-recreational PA were associated with a lower risk of mortality and cardiovascular disease events in individuals from low-income, middle-income, and high-income countries [13]. Jeong compared the impact of leisure-time PA on mortality in primary versus secondary cardiovascular prevention and found that individuals with cardiovascular disease may benefit from PA to a greater extent than healthy volunteers without cardiovascular disease [14]. However, these studies mentioned above did not use continuous monitoring with CIEDs, and the results that were based on questionnaires may have been biased. Recently, Toshihiko found a correlation between PA and all-cause mortality in patients with age > 75 years and pacemakers [12]. However, this population did not have structural heart diseases and were not at risk of sudden cardiac death. And the study did not further clarify the cause of death of the patients. Conraads found that a lower baseline PA was associated with readmission and cardiac death in patients with heart failure [15]. However, the population included in that study was relatively young, and no cut-off value was provided to predict the clinical outcomes. In the present study, the average value of the baseline PA was determined through continuous remote monitoring of ICDs/ CRTDs, the follow-up time was long-term, and the endpoint events were more reliable. The population studied was old people with an average age of nearly 80 years. The results confirmed there was a relationship between PA and cardiac death in patients with age > 75 years and provided a baseline PA cut-off value for clinical use.

According to the current guidelines, the recommendations for older adults are the same as those for middle-aged adults [16]. Due to the effects of diseases and ageing, patients with age > 75 years are limited in their ability to perform PA. Compared with younger adults, old people have a lower level of PA, and it is more difficult for them to reach the recommended level of PA. Picel etal found that frailty was common in old patients with ICDs and associated with mortality [17]. Research results on the benefits of PA in older people has been inconsistent. Cheng concluded that the benefit of leisure-time PA is lower for those aged over 65 years than for those aged younger than 65 years [18]. Another study showed that older people need to perform more moderate and high intensity exercise to gain benefits [19]. Hupin claimed that the recommended level of PA for adults may exceed the standard for older people, and 15 minutes of daily PA may be the best target for older adults [20]. A previous study by Zhao found that the cut-off value of PA in predicting cardiac death in the general population was 7.84% (113 min/day) [21]. Compared with the cut-off value in the general population, that in patients with age > 75 years was lower. However, compared with pacemaker patients without structural heart disease (50 min/day), the cut-off value in patients with age > 75 years implanted with ICDs/CRTDs was higher, which indicates that this cohort of patients with more severe conditions may need more PA to exhibit survival benefits [12]. Therefore, the best recommended value for patients with age > 75 years deserves further discussion. Recently, there have been studies on dose correlation [6, 22–24], and there are still no related studies on the dose relationship in older people with CIED, which can serve as a direction for future research.

### Study limitations

First, our study only included the baseline PA from 30 to 60 days after implantation, longitudinal PA and its changes on the prognosis were not investigated. Moreover, despite of adjustment for multiple covariates, we did not take some important factors such as socioeconomic status and echo indexes besides LVEF and LVEDD. In addition, the use of medical therapy was relatively low though this situation was common in China. Last but not least, this study was limited by a relatively small sample size and retrospective design.

## Conclusions

Daily PA measured by continuous remote monitoring was related to the prognoses of patients with age > 75 years and structural heart diseases and a high risk of sudden death. Daily PA of about 1.5 h was associated with lower all-cause mortality and cardiac death risk in patients with age > 75 years and high risk of SCD with ICDs/CRTDs. PA monitoring may aid in long-term management of those patients.

## Data Availability

The datasets generated and analysed during the current study can be found at Fuwai Hospital but not publicly available due to related regulations. Data and materials are however available from the authors upon reasonable request and with permission of SUMMIT study.

## Abbreviations

PA: Physical activity
SCD: Sudden cardiac death
CIED: Cardiovascular implantable electronic devices
HM: Home monitoring
ICD: Implantable cardioverter-defibrillator
CRTD: Cardiac resynchronization therapy defibrillator
HR: Hazard ratio
CI: Confidence interval
ACEIs: Angiotensin-converting enzyme inhibitors
ARBs: Angiotensin receptor blockers
BMI: Body Mass Index
LVEDD: Left ventricular enddiastolic dimension
LVEF: Left ventricular ejection fraction
NYHA: New York Heart Association
